# Microbial Musings – April 2021

**DOI:** 10.1099/mic.0.001061

**Published:** 2021-05-04

**Authors:** Gavin H. Thomas

**Affiliations:** ^1^​ Department of Biology, University of York, York, UK

The Annual Conference of the Microbiology Society is upon us, and as I wrote these words, our first totally online annual meeting seems to be going down extremely well. Hopefully by the time you read this piece you will have enjoyed a week of super science and be buzzing with new ideas, and if you missed it, then you can still register for the meeting and watch many of the lectures on catch-up next week. Lots of *Microbiology* editors were involved, either in organising sessions or talking themselves, as we aim to make the link between the journal and annual conference (AC) more substantial and look out for themed events at AC2022 to celebrate our 75th Anniversary. As well as some great scientific sessions I particularly enjoyed Elisabeth Bik’s prize lecture as she was nominated by former Editor in Chief Tanya Parish (@ProfTanya13) and myself with respect to her important work on image sleuthing in academic publications. I was delighted at the level of recognition and support for her research integrity work that came from our community of microbiologists after her talk. A most worthy winner.

We have two reviews in this issue, the first from the group of *Microbiology* editor Susanne Gebhard (@GebhardLab) at the University of Bath, UK, who, with her colleagues Timothy Hoffman and Bianca Reeksting (@bjreeksting), have written a very interesting article about the process of bacterial-induced mineral precipitation or BIMP for short [1]. This they define as a bacterial process that indirectly results in mineral formation, which can occur, for example, through the excretion of metabolic by-products that alter the chemical environment. This rather uncontrolled process contrasts to bacterial biomineralization, where the formation of structures like magnetosomes is a highly controlled enzymatic process [2]. While BIMP then seems like a more nonspecific process, the authors bring together all the factors that are known to influence this activity [1]. In doing this they tabulated around 50 different types of minerals that bacteria are known to induce the formation of, including some with the rather anthropomorphic names Bobierrite and Vivianite! They discuss the conditions around which the BIMP crystallization process starts and bacterial cell surfaces turn out to be very suited to this role due to their charge and surface properties that concentrate ions, creating ideal conditions for nucleation. The bacteria themselves need to be alive for BIMP to occur, but in some cases this ends up with them becoming entombed. This lethal problem has led to some interesting solutions and some bacteria shed their S-layers to stop them become too encrusted, a very cool process discovered by the late Terry Beveridge and colleagues at the University of Guelph, Canada, in the 1990s [3]. Some of the applications of BIMP have been featured in the popular press, such as the development of self-healing concrete that contains BIMP-catalysing bacteria [4] and there is clearly lots more to understand about how bacteria have evolved to deal with BIMP and how this could be applied in biotechnology.

The second review provides an extremely thorough overview of the biology of the intracellular lifestyle of the important human pathogen *Mycobacterium tuberculosis* (Mtb) [5]. The review from Leah Rankine-Wilson from the group of Yossi Av-Gay (@yossiavgay) at the University of British Columbia, Canada, summarises the function of all known molecular components of Mtb that are associated with infection and the intracellular survival phenotype. The first stages of this process follow shortly after engulfment by host phagocytes, as the bacteria can use a suite of secreted effector proteins and components of the cell surface to arrest the maturation of phagosomes that would normally result in death. The roles of many of these effectors and drugs being developed to target them are described in detail. I was fascinated by the role of low molecular weight thiols (LMW) like mycothiol (Mtb does not synthesise the classical glutathione molecule) and other unique thiols produced by Mtb that allow it to deal with the oxidative and nitrosative bursts that are turned on during phagocytosis [6]. Despite this extra protective suite of LMW’s, the activation of further oxidative and nitrosative stress by host cells is a direction that is being investigated through new drugs such as Pretomanid [7], which also are thought to be able to reactivate persister cells in the population. The authors finally consider the fate of the infected host cell during the long-term infections that are seen with Mtb, pointing out other stages in this process where antimicrobials might have a chance of getting at these persistent bacteria.

Our popular Microbe Profile series continues this year with *Bdellovibrio bacteriovorus *, a bacterium with an intriguing name and similarly intriguing lifestyle. This predatory bacterium enjoys a highly unusual lifestyle both in the soil and inside the periplasm of prey bacteria, which it then consumes. I wrote about ‘Bdello’ recently in the *Microbial Musings* [8] as we published a nice review considering the evidence for its potential therapeutic use [9] and I highlighted one of the authors of this Profile, Liz Sockett FRS (@Bdello_Lab_Nott) from the University of Nottingham, UK, who has teamed up for this Profile with her long-term Bdello collaborator Andrew Lovering, who works down the road at the University of Birmingham, UK [10]. Bdello’s 3.7 Mb genome reflects its dual lifestyle and the need to survive in soil and contains additional genes that enable its unique recognition, invasion and rapid reproduction in the intraperiplasmic site within its prey [11]. The authors briefly outline some of the key steps in the bacteria’s lifecycle, many of which are really quite unique, for example how the bacterium reseals the host outer membrane after invading and how it can then access cytoplasmic nutrients [12]. One of the important open questions raised by the authors relates to how the bacterium can somehow weigh up the size, and hence nutritional value, of its prey and make a decision as to how many progeny this will support. Finally the bacteria must also sense when the host is exhausted before it triggers cell lysis. Improved genetic tools and advances in fluorescence microscopy have enabled significant advances in understanding this fascinating microbe and there is still a wealth of unusual biological mechanisms to be uncovered.

One paper that caught my eye this month was in our new Microbial Interactions and Communities topic area from Bryden Fields (@BrydenFields) and Ville Friman (@FrimanScience) at the University of York, UK, with Emma Moffat (@thesoundofEmma), and Ellie Harrison (@ellieevolves) at the University of Sheffield, UK [13]. The bacterium *Rhizobium leguminosarum* forms a distinct single species that can establish a characteristic root-nodule nitrogen fixing symbiosis in leguminous plants. However, extensive genome sequencing of this species has revealed significant diversity, such that 18 distinct genospecies have now been defined in a recent work led by my University of York colleague and all-round Rhizobium guru Peter Young [14]. Many early laboratory studies of this symbiosis used single clones to infect the plant, but the biological reality is that nodules form with many distinct genotypes of *R. leguminosarum* on a single plant. The authors set out to investigate using a set of 12 *R *. *leguminosarum* strains how symbiont diversity influences the productivity of the rhizobia-legume symbiosis. Using different multi-strain inoculations of plants and by measuring plant performance, they concluded that there was only a weak positive correlation between symbiont genotype diversity and plant performance. This is consistent with there being no additional costs to the plant in hosting diverse *R. leguminosarum *, which could impact on bio-inoculant design that usually only contains a handful of strains and often not enough diversity to outcompete the range of natural isolates in the soil. Bryden also presented this work this week at AC2021 in the Genetics and Genomics symposium, so many of you will have heard this explained much better than my attempt at a summary presented here!

By now, regular readers of the *Microbial Musings* will be familiar with the explosive cell lysis phenomenon used by some bacteriophage to exit their hosts, from the papers of Cynthia Whitchurch’s group (@Cwhitch), including a paper published in *Microbiology* early this year [15, 16]. Now the group, from the iThree Institute (@ithreeinst), University of Technology Sydney, Australia and the Quadram Institute (@TheQuadram) in Norwich, UK, including Laura Nolan (@LauraNolanLab), are looking in more detail at the formation of membrane vesicles (MVs) during this process during infection of *Escherichia coli* with T4 or T7 bacteriophage [17]. Using a combination of live cell imaging techniques, including some beautiful super-resolution microscopy, Pappu Mandal and Giulia Ballerin produce some stunning images showing the process of vesicle formation. Using these methods they demonstrate that the *E. coli* phage induce similar explosive cell lysis to that seen with their *Pseudomonas* cousins, which also results in extensive MV formation. Curiously, both the kinetics of killing of the *E. coli* population and the types of MVs produced differ during infection with either T4 or T7, with T7 infection resulting in larger MVs. They could also observe MV formation through membrane blebbing after infection but before cell lysis. While the explosive lysis undoubtedly results in MV formed from the inner membrane, further work needs to clarify the contribution of MV comprised of inner and outer membrane that are liberated during these different processes. Overall, they argue that the production of MVs in real environments, such as seawater where lytic bacteriophage are massively abundant, is likely to be highly significant.

One piece of journal-related news this month is the Microbiology Society switching to a continuous publication model. You might have noticed that instead a holding articles for compilation into an issue at the end of the calendar month, articles are now published immediately in an Open Issue, which are then compiled into the final issue at the end of the month.

Finally, as COVID-19 levels in the UK are now down to the lowest in Europe, and many of us, including myself this week, have been vaccinated, lets spare a thought for our colleagues in India, Brazil and other countries where the virus is currently causing massive loss of life and distress for thousands of people. As we heard form Emma Thomson (@emcat1) at AC2021, the virus isn’t going away, and new variants are not currently becoming any less virulent, suggesting that global monitoring and intervention is essential for keeping the disease under control.

Gavin H. Thomas

Department of Biology, University of York, UK, YO10 5YW.
